# Living Memory Home for Dementia Care Pairs (LMH-4-DCP): study protocol for a pilot randomized trial of a web-based reminiscence intervention for family caregivers and persons with dementia

**DOI:** 10.1186/s40814-026-01781-2

**Published:** 2026-04-14

**Authors:** Francesca B. Falzarano, Annabelle Greenfield, Sydney C. Saviano, Sindhu Kolla, Sosi Korian, Francesco Osso, Joseph Miller, Paul K. Maciejewski, Heather E. Whitson, Holly G. Prigerson

**Affiliations:** 1 Leonard Davis School of Gerontology, University of Southern California, Los Angeles, CA USA; 2 Department of Radiology, Cornell Center for Research On End-of-Life Care, Weill Cornell Medicine, New York, NY USA; 3 Duke Aging Center and Duke/University of North Carolina Alzheimer’s Disease Research Center, Duke University School of Medicine, Durham, NC USA; 4 Geriatrics Research Education and Clinical Center (GRECC), Durham VA Medical Center, Durham, NC USA

**Keywords:** Alzheimer’s disease and related dementias, Reminiscence therapy, Pre-death grief, Dementia family caregiving, Relationship quality, Dyadic interventions

## Abstract

**Background:**

Family caregivers provide most of the emotional, physical, and logistical support for people living with Alzheimer’s disease and related dementias (ADRD). As the caregiving role intensifies with advancing illness, family caregivers navigate the cognitive and functional decline of the care recipient. They also must reconcile the shift from a mutual relationship to one of a primary caregiving role. The unique nature of ADRD makes pre-death grief highly prevalent among family caregivers. Reminiscence therapy, which uses memory prompts to evoke meaningful past experiences, has shown promise in improving psychosocial outcomes for individuals with dementia. Its potential to benefit caregivers through strengthened emotional connections, shared meaning, and mutual engagement among the dyad, however, has been largely underexplored. Guided by the Interdependence Model of Communal Coping and the Micro-Sociological Theory of Adjustment to Loss, the current study aims to assess the feasibility and acceptability of Living Memory Home for Dementia Care Pairs (LMH-4-DCP), a reminiscence-based online intervention for family caregivers and their care-recipients.

**Methods:**

This multisite pilot randomized controlled trial (RCT) will evaluate the feasibility, acceptability, and preliminary efficacy of LMH-4-DCP. Seventy ADRD family caregivers will be targeted for randomization to a 1:1 ratio to the intervention (*n* = 35) or attention control (*n* = 35) conditions and will participate for 2 weeks. Recruitment will be facilitated by the project’s two study sites located in urban metropolitan areas of the USA. Primary feasibility outcomes include recruitment, retention, and completion rates, website usability (e.g., perceived usefulness and ease of use), and intervention satisfaction. Exploratory analyses will be conducted to assess the preliminary efficacy of LMH-4-DCP in reducing pre-death grief and improving relationship quality. Outcomes will be measured using validated questionnaires at baseline and 2-week follow-up, along with semi-structured interviews with LMH-4-DCP participants.

**Discussion:**

This pilot trial will offer foundational evidence regarding the feasibility and acceptability of the LMH-4-DCP intervention. Study findings will guide intervention refinements and inform the design of a larger-scale, fully powered RCT to evaluate its efficacy in reducing pre-death grief and enhancing relationship quality among ADRD family caregivers.

**Trial registration:**

Clinicaltrials.gov: NCT06225986, Registered: January 9, 2024, ClinicalTrials.gov; https://clinicaltrials.gov/study/NCT06225986?term=living%20memory%20home&rank=1

**Supplementary Information:**

The online version contains supplementary material available at 10.1186/s40814-026-01781-2.

## Background and rationale

Family caregivers provided the majority of emotional, physical, and logistical support for more than seven million individuals living with Alzheimer’s disease and related dementias (ADRD) in the USA [1]. In 2024, an estimated 11 million people provided unpaid assistance to a family member or friend with ADRD, underscoring the important role of family caregivers in managing the health of persons with dementia (PwD). Despite their important contributions, the stress associated with ADRD care provision exacerbates risk for adverse physical, psychosocial, and economic outcomes. Beyond the demands of daily care, ADRD family caregivers often experience significant psychological distress stemming from the progressive cognitive, emotional, and interpersonal losses associated with disease advancement.

ADRD caregiving is marked by cumulative losses—both experienced and anticipated—which contribute to complex grief about experiences in the past, present, and imagined future. In addition to anticipating the death of the care-recipient, ADRD caregivers uniquely face a progressive loss of emotional reciprocity, connection, and mutual recognition [2]. The construct of relational deprivation describes this transition from a reciprocal, mutually supportive relationship to a predominantly unilateral caregiving role [3, 4]. These interpersonal losses intensify pre-death grief—a form of mourning characterized by feelings of longing, yearning, and role confusion that occurs prior to the physical death [5, 6]. While the care-recipient remains physically present, pre-death grief in ADRD caregivers often leads to psychosocial disruptions comparable to those observed in bereavement [7].

In non-ADRD caregiving populations (e.g., cancer), pre-death grief is a strong predictor of adverse mental health outcomes, including depression, anxiety [8], poor coping [9, 10], diminished quality of life [11], and impaired end-of-life decision-making [7]. A growing body of evidence suggests that ADRD pre-death grief may be even more intense than post-death grief [12, 13] and is associated with increased risk for Prolonged Grief Disorder (PGD) [14], which is a well-established predictor of suicidal ideation and a host of adverse outcomes [15]. Despite its clinical relevance, pre-death grief remains largely absent from prevailing models of dementia caregiver stress.

Reminiscence therapy (RT) is a person-centered intervention that uses cues to evoke autobiographical memory recall for significant life events [16]. Evidence from a Cochrane review [17] suggests that RT shows potential to support quality of life, mood, communication, and possibly cognition in PwD. Findings on RT’s effectiveness remain largely inconsistent, with considerable heterogeneity in delivery format, context, duration, and facilitation [18]. RT interventions have been mostly tested in long-term care settings using group-based formats, with limited focus on caregiver outcomes or dyadic delivery approaches. While family caregivers shoulder 83% of the assistance provided to PwD [1], there are few interventions targeting RT in home-based settings or via web-based delivery formats [19]. To date, research on digital reminiscence tools for use in the home, particularly those involving family caregivers as facilitators, remains limited.

Using RT to facilitate storytelling and preserve meaningful memories in PwD has demonstrated potential for improving psychosocial outcomes among family caregivers. The Interdependence Model of Communal Coping suggests that shared, meaningful activities within caregiving dyads can alleviate stress and enhance relational quality [20]. Consistent with this framework, research indicates that engaging in joint activities can reduce adverse mental health outcomes by reinforcing emotional connection, meaning-making, and mutual engagement [21]. RT-based activities may support cognitive reframing by helping caregivers focus on positive relational experiences—a process linked to increased relational closeness, even amid cognitive decline—and may play a critical role in mitigating pre-death grief [22].

The Micro-Sociological Theory of Adjustment to Loss [23] further emphasizes the need to address psychosocial voids—such as diminished belonging, appreciation, and emotional reciprocity—to foster adaptive coping and promote healthy bereavement adjustment. In the pre-death context of ADRD, such voids often emerge as anticipatory and ambiguous losses, as caregivers confront the erosion of mutual recognition and shared identity. Together, these theoretical frameworks highlight the value of strengths-based, meaning-centered interventions that sustain relational connection. Structured RT activities offer a practical means to promote meaningful, reciprocal engagement within caregiving dyads—helping to preserve emotional bonds and reduce caregiver distress, even in the context of progressive cognitive decline.

Building on this foundation, the current study aims to adapt an existing bereavement tool into a dyadic, RT–based pre-death digital intervention for ADRD caregiving dyads. The *Living Memory Home* (LMH) is a digital platform developed to support bereaved individuals through structured writing, reflection, and imagined dialogue activities. In a pilot study (*n* = 100), participants frequently exceeded minimum usage expectations and reported strong emotional engagement [24]. Notably, LMH use over 1 week was associated with a significant reduction in PGD symptoms (PG-13; *p* = 0.02). Qualitative feedback included suggestions to develop a version of LMH for use before their loved one’s death. This feedback, along with growing evidence that anticipatory grief among ADRD caregivers is widespread and often more intense than post-death grief [25–27], led to a pre-death adaptation of LMH using RT as a digital intervention for dementia caregiver–care-recipient dyads (hereafter referred to as “Care Pairs”).

## Aims and objectives

The overarching aim of this study is to adapt and pilot test a web-based intervention—Living Memory Home for Dementia Care Pairs (LMH-4-DCP)—for ADRD care pair dyads. The intervention incorporates reminiscence-based activities to promote collaborative engagement and assist caregivers in eliciting and preserving the life story of the PwD. Specifically, the current study aims to adapt the existing LMH bereavement tool [24] to form the LMH-4-DCP web-based RT platform for family caregivers and individuals with early-to-moderate dementia.

### Primary objective

To evaluate the feasibility and acceptability of the LMH-4-DCP intervention among ADRD family caregivers.

### Secondary objective

To assess the preliminary efficacy of LMH-4-DCP in reducing pre-death grief and improving relationship quality among care pairs.

### Exploratory objectives

1. To explore potential mediators, such as role confusion and sense of belonging, that may influence the relationship between LMH-4-DCP engagement and caregiver outcomes (pre-death grief and relationship quality).
2. To generate preliminary effect size estimates to inform sample size calculations for a future fully powered randomized controlled trial (RCT).

### Progression to full trial

The key indicator of study success and progression will be (a) recruitment, (b) retention, and (c) platform usability. Progression from feasibility to a future definitive RCT would be warranted based on the *Go/No-Go *criteria detailed below:

- *Go:* (a) recruitment of at least 70% of the targeted sample size (total *N* = 70, i.e., 35 participants per arm), (b) < 40% attrition rate (attrition = no follow-up assessment completed), and (c) a minimum score of 68 on the System Usability Scale (SUS).
- *No-Go:* (a) recruitment below 70% of planned, (b) attrition rate > 50%, and (c) SUS < 68.

If recruitment, retention, and/or usability are between stop-and-go criteria (i.e. recruitment between 70 and 100% of planned; attrition between 40 and 50%; SUS between 50 and 67 [28]): significant remedial action would need to be taken for the study to progress to a full RCT. Such approaches could include additional recruitment methods, study sites, or alterations to the intervention, and/or the digital platform based on qualitative feedback from study participants.

## Methods

This protocol paper follows the Standard Protocol Items: Recommendations for Interventional Trials (SPIRIT) and Consolidated Standards of Reporting Trials (CONSORT) reporting guidelines for feasibility studies [29, 30]. The order of the items has been modified to group similar items. See Supplementary material S1 for SPIRIT checklist.

### Study design

This is a multisite, participant-blinded, pilot RCT designed to assess feasibility, acceptability, and preliminary efficacy of the LMH-4-DCP intervention. A total of 70 ADRD family caregivers will be randomized to either the intervention group or an attention control group and will be enrolled in the study for a 2-week duration. Intervention participants will receive access to the full LMH-4-DCP platform, while control participants will access a restricted, non-RT version (described below).

All study activities, including informed consent, intervention delivery, and outcome assessment, will be conducted virtually. Data will be collected using Research Electronic Data Capture (REDCap), a secure, Health Insurance Portability and Accountability Act (HIPAA)-compliant electronic data management system, at baseline and 2 weeks post-intervention. Intervention participants will also be invited to complete an optional semi-structured interview about their experience with the LMH-4-DCP intervention.

### Study setting

The study will be conducted virtually but coordinated through two academic institutions: the primary site at Weill Cornell Medicine (WCM, New York, NY, USA) and a secondary site at the University of Southern California (USC, Los Angeles, CA, USA).

### Eligibility

#### Inclusion criteria

Participants must

1. Be the primary caregiver of a community-dwelling family member or friend with early-to-moderate ADRD
2. Be 18 years of age or older
3. Reside in the USA
4. Speak English fluently
5. Have internet access and basic digital proficiency
6. Be willing and able to provide informed consent.

#### Exclusion criteria

Individuals will be excluded if they

1. Are under 18 years of age
2. Are not the primary caregiver of a community-dwelling person with ADRD
3. Do not speak English fluently
4. Do not reside in the USA
5. Lack internet access or ability to use web-based platforms
6. Have cognitive impairments that interfere with informed consent or participation

### Sample size

This study will enroll 70 family caregivers of individuals with ADRD, with participants randomized in equal numbers (*n* = 35 per group) to the intervention or an attention control condition. The primary goal of this pilot study is to establish the feasibility and acceptability of LMH-4-DCP and to obtain effect size estimates to inform the design of a future definitive RCT; accordingly, the study is not powered to detect clinically meaningful differences in outcomes. A sample size of 35 per arm (*N* = 70) is at the upper end recommended for feasibility trials [31], and provides adequate precision for estimating key feasibility parameters (recruitment, retention/attrition, and platform usability) needed to inform prespecified progression (Go/No-Go) criteria for a future definitive RCT. For example, *N* = 70 allows for feasibility proportions such as retention to be estimated with sufficient precision to support decision-making regarding progression to a full-scale RCT. This approach is consistent with methodological guidance for pilot and feasibility studies, which emphasizes estimation of feasibility metrics over formal hypothesis testing or power-based sample size determination [31–34].

### Intervention description

The development of LMH-4-DCP followed a user-centered design framework to ensure the intervention aligned with the needs, preferences, and experiences of both caregivers and care-recipients. Between March and May 2024, semi-structured interviews were conducted with 32 stakeholders, including 22 ADRD family caregivers and 10 subject matter experts (e.g., dementia care coordinators, social workers). Participants reviewed an early prototype of the LMH-4-DCP platform and proposed website components. Feedback related to content, design, usability, and functionality was used to iteratively refine the platform. This stakeholder-informed development process ensured the intervention was contextually relevant and acceptable before initiating the RCT phase of the study. Based on this formative feedback, the finalized LMH-4-DCP platform includes several core and RT-specific features.

Participants in both study arms will be asked to log in and complete intervention activities three times per week over 2 weeks. Upon first login, all users will receive a guided onboarding experience that introduces platform features and facilitates initial engagement. As part of this onboarding, participants will select one of 12 virtual home environments—ranging from imaginative (e.g., spaceship, castle) to realistic (e.g., beach house, cabin, city apartment) settings—which serve as the visual theme and backdrop for all subsequent activities. This personalization feature is intended to enhance emotional comfort, narrative immersion, and sustained user engagement.

### Study conditions

Participants will be randomly assigned to either the intervention group or an attention control group. Each group will access a version of the LMH-4-DCP platform tailored to their assigned condition, allowing for consistent engagement while isolating the impact of reminiscence-based content. See Table 1 for a detailed comparison of LMH-4-DCP features by study condition.

**Table 1:** LMH-4-DCP feature breakdown of reminiscence and non-reminiscence-based activities for intervention and control group participants, respectively

Feature name	Description	RT	Non-RT
**Reminiscence room**			
Memory lane	A digital scrapbook where dyads can upload photos and are asked to reflect and reminisce on experiences and events. Family caregivers will facilitate uploading photos and creating captions with the PwD	•	
Wall of fame	Dyads upload and choose photos and respond to generated prompts. Dyads will be focused on both the PwD’s meaningful accomplishments (e.g., graduation) and/or significant people in the PwD’s life (e.g., family photos). These photos will be virtually posted on the wall of their ‘Reminiscence Room’	•	
**Writing room**			
This is Your Life	Caregivers will serve as biographers, interviewing the PwD using 55 prompts to document their major milestones, ultimately resulting in a chronological ‘Life Story’ across early childhood (14 prompts), adolescence (9 prompts), young (13 prompts), middle (6 prompts), and older adulthood (13 prompts). User entries will be compiled to produce a “Book of Your Life,” chronologically presenting major “chapters” of the PwD’s life. Users have the option to export and save as PDF	•	
Journaling (RT: intervention group)	The journal will contain structured prompts that will be used for caregivers to facilitate and document the PwD’s current feelings and experiences, and hobbies, as well as reminiscence-based prompts that focus on recalling past events • 35 RT prompts • 25 non-RT prompts	•	
Journaling (non-RT: control group)	25 structured prompts will be used for participants to reflect and document the PwD’s current feelings and experiences, hobbies, and/or any other relevant circumstances or scenarios occurring in the present		•
**Reading room**			
Resource dictionary	A comprehensive list of formal home and community-based organizations (HCBOs) both locally and nationally. Includes organizations, helplines, and supportive communities		•
Tips and tricks	Informal sources of support, helpful tips, education, and information—including Podcasts, YouTube videos, social media accounts, popular press articles, etc.—to help caregivers more easily navigate their role		•
Information station	Access to latest scientific research and advancements via articles, publications, and help guides/toolkits in ADRD, family caregiving, etc.		•

Note:* RT *reminiscence therapy, *ADRD *Alzheimer’s disease and related dementias

#### Intervention group

Intervention participants will have access to the full LMH-4-DCP platform, which includes three interactive features designed to support reflection, storytelling, and connection among care pairs. (1) The *Reminiscence Room* features RT-based activities, including a digital scrapbooking tool to facilitate collaborative memory-sharing and storytelling. (2) The *Writing Room* offers structured reflective writing exercises based on reminiscence prompts (e.g., “Describe a favorite memory from your childhood”; “Describe a significant milestone or achievement from your lifetime”) to support life review and narrative meaning-making. (3) The *Reading Room* provides access to a curated selection of caregiver resources, including formal educational content from national organizations and other resources such as websites, blogs, and YouTube videos.

#### Attention control comparator

Participants in the control group will access a restricted version of the LMH-4-DCP platform that excludes RT-based features. Control activities are designed to match the intervention condition in terms of time commitment and interface interaction, but do not incorporate reminiscence or life review elements. Participants in this group will (1) personalize the user interface by selecting a virtual home environment; (2) complete journal entries in the *Writing Room* using neutral (non-RT), present-focused prompts (e.g., “What’s on your mind today?”; “If you could do anything now, what would it be?”); and (3) access the same educational caregiving resources via the *Reading Room* as the intervention group. This control condition was designed to ensure comparable engagement across groups while enabling the isolation of RT-specific effects on primary outcomes, including pre-death grief and relationship quality.

### Recruitment

Sample recruitment will be facilitated by the project’s two study sites using a multi-pronged recruitment strategy, including (1) posting study flyers in high-traffic areas in New York City, Southern California, and Seattle, Washington; (2) study announcements made via the Duke University–University of North Carolina Alzheimer’s Disease Research Center (Duke/UNC ADRC); (3) ResearchMatch.org, a National Institutes of Health (NIH)-funded online registry that connects research volunteers with IRB-approved studies; and (4) social media advertising on platforms such as X (formerly Twitter), Facebook, and Instagram.

### Ethics

This study was approved by WCM’s Institutional Review Board (IRB) on August 13, 2024, and received single IRB (sIRB) approval on October 23, 2024 (sIRB#: 23–07026251). The trial is registered on ClinicalTrials.gov (NCT06225986). IRB approval letters for the original and amended protocols can be found in Supplementary materials S2 and S3.

#### Informed consent

Once eligibility is confirmed, trained, IRB-approved research assistants (RAs) will obtain informed consent via a Zoom or telephone session. During the session, the RA will review the study in detail and invite participants to ask questions at any time. Participants will be informed of the purpose of the study, its funding sources, who is conducting the research, and how they were selected.

The RA will explain what participation involves, including time commitment, tasks, potential risks and benefits, and any associated costs. They will emphasize the importance of confidentiality and explain participants’ rights, including the right to withdraw at any time without penalty. As consent is an ongoing process, participants will be promptly informed of any protocol changes that may affect their willingness to continue in the study. The consent form will also request permission to audio-record interview sessions; participants who decline may still take part, with staff taking written notes instead.

Participants interested in proceeding will be guided by the RA to sign and date the consent form electronically. All participants will receive a signed copy of the consent form, countersigned by the RA, for their records. Per guidelines set forth by the International Council for Harmonisation of Technical Requirements for Registration of Pharmaceuticals for Human Use on Good Clinical Practice (ICH-GCP) and applicable local regulations, documented informed consent (written or electronic) is required before study participation (see Supplementary material S4 for Informed Consent Form).

All study personnel are trained in the protection of human subjects, confidentiality, and recruitment protocols. Data collection, sharing, and storage will comply with HIPAA and IRB regulations. Each enrolled participant will be assigned a unique study identifier. All data will be stored in a secure, password-protected REDCap database accessible only to authorized personnel. To maintain confidentiality, all datasets will remain de-identified throughout the study, including during analysis and dissemination.

### Data management

All study data will be stored on a secure, password-protected server housed at the primary study site, with access restricted to authorized study personnel. REDCap will be used for participant tracking, survey data collection, and adverse event reporting for all enrolled subjects. Supported by WCM’s Clinical and Translational Science Center (CTSC), REDCap is a secure, web-based data management platform that enables the development of customized data management systems with web-based entry forms, reporting tools, and robust security features—including user- and role-based access controls, institutional LDAP (Lightweight Directory Access Protocol) authentication, and a complete audit trail of data entry and export activities.

REDCap is hosted on CTSC-managed servers, which are backed up nightly and support encrypted (SSL-based) connections. The platform is maintained nationally through a multi-institutional consortium led by the Vanderbilt University Clinical and Translational Science Award (CTSA) program.

### Randomization

Following informed consent, participants will be randomized to either the intervention or attention control group using REDCap’s randomization module before completion of the baseline survey. To enhance internal validity and reduce potential confounding, we implemented a stratified permuted block randomization design. Randomization was conducted by a study RA using a custom R script that generated 2:2 allocations within blocks of four, stratified by participant gender and race. This approach ensured demographic balance across study arms while accommodating the target sample size (*N* = 70 dyads).

The script used an unset random seed to preserve allocation unpredictability across runs. Randomization tables were generated for each of 20 possible gender-by-race strata and exported as a CSV file, which was then uploaded to REDCap’s secure randomization module. Once uploaded, the allocation table is locked—no longer visible or modifiable by study personnel. Participants will be assigned to conditions in real time by study staff via REDCap after consent is obtained and stratification variables (gender, race) are entered. This process preserves allocation concealment and ensures methodologically sound group assignment.

### Blinding

Participants will remain blinded to group allocation throughout the study, will not have access to any materials or documentation that discloses their assignment, nor will they be unblinded under any circumstances. The informed consent process will explain that all participants will use the LMH-4-DCP platform, but it will not disclose which features differ by study condition, thereby preserving blinding.

Both groups will undergo similar procedures: participants will complete the same baseline survey, receive a condition-specific registration link, and use the platform over the same 2-week period. Each group will also receive training videos tailored to their assigned version of the platform. Training materials for the control group are designed to conceal the absence of any features, and participants will not have access to the instructional content of the other group.

### Assessments and outcomes

**Fig. 1:**
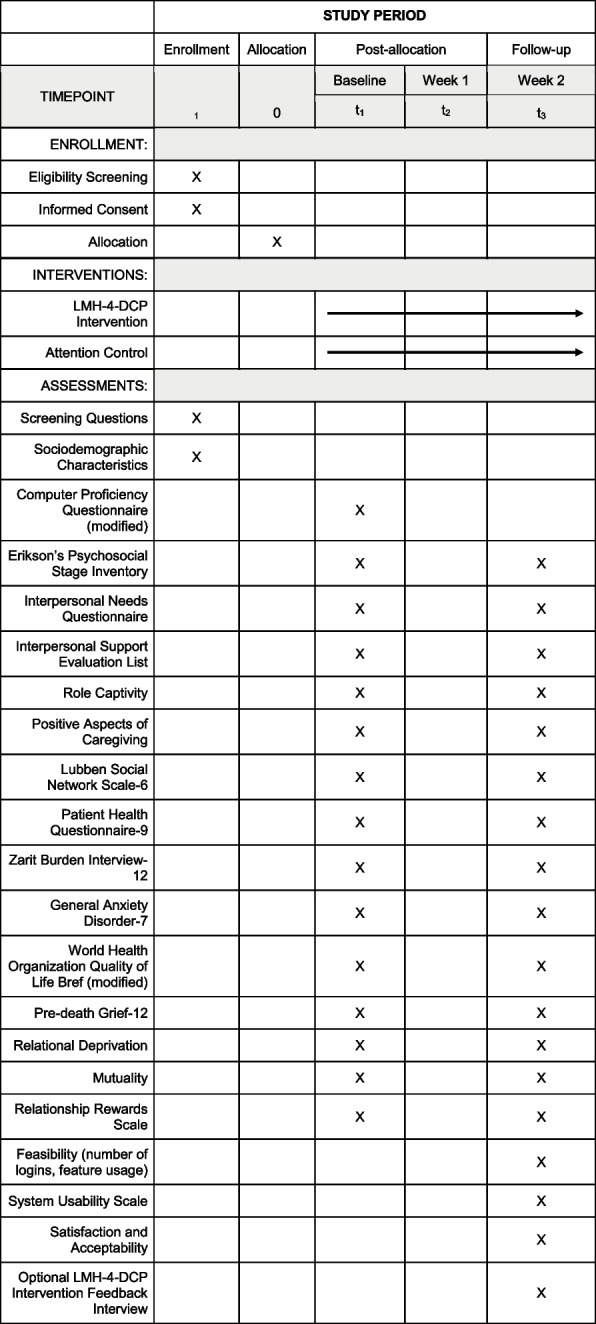
SPIRIT Figure: LMH-4-DCP Trial Assessments and Timepoints

Prospective participants will be emailed a link to complete a brief eligibility screening on REDCap. The screening will include items related to basic demographic questions (e.g., age, education) and caregiving-specific questions to determine eligibility based on the inclusion/exclusion criteria described above. Study assessments will be administered electronically via REDCap at two time points: baseline and 2-week follow-up. Intervention participants will also have the option to complete a post-intervention feedback interview via Zoom or telephone. A description of measures can be found in Table 2 and a breakdown of assessments by time point is presented in Fig. 1. Participants will receive $50 for each completed survey and an additional $20 for the optional interview, for a maximum total compensation of $120. All payments will be distributed as Amazon.com digital gift cards.

**Table 2:** Description of Survey Instruments

Domain	Measure	Description
Sociodemographic characteristics	N/A	Age, sex, race/ethnicity, education, geographic location, income, CR functional status, and dementia-related problem behaviors
Technology proficiency	Computer Proficiency Questionnaire (modified) [35]	16-item scale assessing older adults’ proficiency with technology use. Sample items begin with the stem: “I can”… followed by statements such as “Find information about my hobbies and interests on the Internet” and “Use search engines (e.g., Google, Bing).” Items are rated from 1—never tried to 5—very easily; higher scores reflect greater technology proficiency. Cronbach’s α =.98
Identity confusion	Erikson’s Psychosocial Stage Inventory: Identity Subscale (Modified) [36]	12-item questionnaire assessing identity-related challenges based on Erik Erikson’s stages of psychosocial development. Sample items include” “I’ve got a strong sense of what it means to be me” and “I’ve got it together.” Response options range from 1—hardly ever true to 5—almost always true. Cronbach’s α =.85 [37].
Belonging/ appreciation	Interpersonal Needs Questionnaire [38]	15-items assessing the extent to which individuals feel connected to and valued by others across two subscales: Thwarted Belongingness (9-items; α =.85) and Perceived burdensomeness (6-items; α =.89). Items are rated on a Likert-scale ranging from 1—not at all true for me to 7—very true for me. Cronbach’s α =.85 for total scale
Role confusion	Role Captivity Scale [3]	3-items measuring role confusion and the psychological impact of feeling confined to a role. Sample items include: How much do you: “Feel trapped by your relative’s illness?” Response options are rated on a Likert scale ranging from 1-Not at all to 4-Very much. Cronbach’s α =.83
Rewards	Positive aspects of caregiving [39]	11-item questionnaire evaluating the positive experiences and rewards associated with caregiving. Items begin with the stem: “Providing help to my care-recipient has” with sample items including “Made me feel more useful” and “Given more meaning to my life.” Response options range from 0-Disagree a lot to 4-Agree a lot. Cronbach’s α =.92
Social support	Lubben Social Network Scale-6 (LSNS-6) [40]	6-item inventory assessing the availability and quality of social relationships and support systems across: (1) family (3-items; e.g., “How many relatives do you see or hear from at least once a month?”) and (2) friends (3-items; “How many friends do you feel close to such that you could call on them for help?”). Response options range from 0—none to 5—nine or more. Cronbach’s α =.75
	Interpersonal Support Evaluation List (ISEL) [41]	40-item questionnaire measuring perceptions of social support across four subscales: (1) tangible (10-items; α =.79); (2) appraisal (10-items; α =.81); (3) self-esteem (10-items; α =.70); and (4) belonging (10-items; α =.76). Response options range from 0—definitely false to 3—definitely true. Cronbach’s α for the total scale =.92
Burden	Zarit Burden Inventory (ZBI-12) [42]	12-item measure of caregiver burden related to emotional, physical, and financial stress. Sample items begin with the stem “How much do you feel…” followed by statements such as: “Strained/tense around your CR” and “You could do a better job in caring for your CR.” Items are rated on a scale from 0—never to 4—nearly always. Cronbach’s α =.88
Depression	Patient Health Questionnaire-9 (PHQ-9) [43]	9-item scale assessing the severity of depressive symptoms occurring within the past 2 weeks. Sample items include “Little interest or pleasure in doing things” and “Feeling down, depressed, or hopeless.” Response options range from 0—not at all to 3—nearly every day. Cronbach’s α =.89
Anxiety	Generalized Anxiety Disorder-7 (GAD-7) [44]	7-item scale assessing anxiety symptoms within the last 2 weeks such as “Feeling anxious, nervous, or on edge”; “Not being able to control or stop worrying.” Response options range from 0—not at all to 3—nearly every day (α =.92)
Quality of life	WHO-QoL Bref (modified) [45]	Five items assessing overall quality of life (1 item; 1—very poor to 5—very good); life satisfaction (2-items; 1 = not at all to 5 = an extreme amount); and satisfaction with personal relationships (2-items; 1 = very dissatisfied to 5 = very satisfied).^+^
*Primary outcomes*		
Feasibility	Recruitment and study completion	Recruitment: proportion of participants who (1) consented; (2) declined participation; (3) did not meet eligibility criteria; Completion rates, attrition rates
Usability	System Usability Scale (Modified) [46]	10-item assessment of website usability. Sample items include “I think that I would like to use LMH-4-DCP frequently” and “I thought the LMH-4-DCP system was easy to use, with responses ranging from 1—strongly disagree to 5—strongly agree. Cronbach’s α =.92 [47]
Acceptability	Satisfaction, acceptability	Survey and open-ended items assessing usability, engagement, acceptability, suggestions for improvement
*Secondary outcomes*		
Pre-death grief	PG-12 [5, 48]	12-item measure assessing frequency of distressing grief symptoms related to yearning, bitterness, interpersonal disengagement, meaninglessness. Sample items include “In the past month, how often have you felt yourself longing or yearning for (patient) to be healthy again?” Response options range from 1—not at all to 5—several times a day/overwhelmingly. Cronbach’s α =.81–.88
Relationship quality	Relational deprivation [3]	6-item inventory measuring the perceived relationship loss with the care-recipient across two subscales: (1) deprivation of intimate exchange (3-items, α =.77; e.g., “How much have you lost: Being able to confide in your relative?”); (2) deprivation of goals and activities (3-items, α =.67; e.g., “How much have you lost: A chance to do some of the things you planned?”). Responses range from 1—not at all to 4—completely
	Mutuality [49]	15-item questionnaire assessing the reciprocity in the caregiver-CR relationships. Sample items include “To what extent do the two of you see eye to eye” and “How close do you feel to him or her.” Response options range from 0—not at all to 4—a great deal. Cronbach’s α =.90
	Relationship Rewards Scale [50]	8-item questionnaire evaluating the perceived benefits derived from the caregiver’s relationship with the care-recipient prior to (α =.84) and after (α =.81) illness onset. Responses range from 1—never to 4—always

Note: *CR* Care-recipient; ^+^Cronbach's alphas not reported for two-item measures; Pearson’s correlation coefficients will be examined to assess the strength of the relationship between items

#### Primary outcomes

##### Feasibility

Feasibility will be assessed through multiple metrics across recruitment, retention, and platform usability. Recruitment feasibility [51] will be evaluated by tracking the number and proportion of family caregivers who (1) consent to participate, (2) decline participation, and (3) are ineligible for study participation at screening. Recruitment data—including the number of individuals who express interest, the number agreeing to participate, and the number who provide consent—will be recorded in REDCap using timestamped research logs and used to calculate consent rates (e.g., the number consented divided by the number interested). Completion and attrition rates will be examined for the entire sample as well as by study arm and will be analyzed by calculating the number of completed assessments per participant divided by the total number (*n* = 2) of assessments. A designated data manager will oversee the collection, monitoring, and reporting of feasibility-related data.

Website feasibility will be examined through backend usage analytics, including the number of logins, time spent in each feature, and overall activity patterns. Additionally, participants will complete a 10-item modified version of the SUS, which captures participants’ perceptions of usefulness, ease of use, and satisfaction. Items include statements such as “I think I would like to use LMH-4-DCP frequently” and “I thought the LMH-4-DCP system was easy to use,” rated on a 5-point Likert scale (1 = strongly disagree to 5 = strongly agree). Scores will be converted to a 0–100 scale, with higher scores indicating greater usability. These metrics will inform the overall feasibility of recruitment, engagement, retention, and website procedures, as well as modifications needed for a future large-scale trial.

##### Acceptability

Acceptability will be assessed at the 2-week follow-up using an 8-item survey designed to assess user satisfaction, perceived usefulness, and ease of use. Participants will rate their agreement with statements such as, “I enjoyed my experience using the LMH-4-DCP application” and “I found the application’s tutorials and instructions easy to follow.” Items are rated on a 5-point Likert scale (1 = strongly disagree to 5 = strongly agree). In addition, participants in the intervention group will complete five open-ended questions to provide qualitative feedback on their experience, specifically related to aspects of the intervention perceived as engaging, helpful, or in need of improvement. Sample prompts include: “Which LMH-4-DCP features were most valuable and why?” 

#### Secondary outcomes

##### Pre-death grief

Grief will be assessed using the 12-item pre-death version of the PGD (PG-12-R) [5, 48] measure, which asks caregivers to report on the frequency of grief symptoms (e.g., yearning, bitterness, disengagement, meaninglessness) due to their care-recipient’s terminal condition (e.g., “In the past month, how often have you felt yourself longing or yearning for (patient) to be healthy again?”). Responses are rated on a 5-point Likert scale ranging from 1 = not at all to 5 = several times a day or overwhelmingly. Total scores range from 12 to 60, with higher scores indicating greater intensity of pre-death grief (Cronbach’s α = 0.81–0.88).

##### Relationship quality

Three validated instruments will be used to assess different dimensions of relationship quality between the caregiver and the care recipient. The six-item* Relational Deprivation Scale *[3] measures perceived relational loss resulting from the care-recipient’s illness. Each item begins with the stem “How much have you lost…” followed by statements such as “Being able to confide in your relative” and “A chance to do some of the things you planned.” Response options range from 1 = Not at all to 4 = Completely, with higher scores indicative of poorer relational quality (α = 0.67–0.77). 2) The 15-item *Mutuality Scale *[37] will be also administered to evaluate the positive aspects of caregiving. Sample items include: “To what extent do the two of you see eye to eye?” and “How close do you feel to him or her?” Response options range from 0 = not at all to 4 = a great deal, with higher scores reflecting greater levels of mutuality (α = 0.90). The 8-item *Relationship Rewards Scale* asks caregivers to report on the quality of their relationship before and after the care-recipient's diagnosis of ADRD. Responses range from 1 = Never to 4 = always, with higher scores indicating greater relational rewards (α = 0.81–0.84) [50].

### Retention and adherence

A structured retention and adherence protocol will be implemented at the time of participant enrollment. Research staff will be trained to respond sensitively to participants’ questions and needs throughout the study, including addressing issues such as fatigue, emotional distress, and barriers to engagement. The WCM research team will track engagement using back-end analytics, including real-time login frequency and feature usage, to guide the content of 1-week check-in contacts.

Participants will receive a 1-week check-in from a study team member via phone or email. Participants who have logged in will be contacted to assess their experience, address any concerns, and offer optional one-on-one support via Zoom or telephone. Participants who have not yet logged in will receive a reminder email with encouragement to engage, followed by weekly reminders for up to 3 weeks. If there is no response after that period, they will be considered lost to follow-up. This structured follow-up is designed to enhance adherence by reminding participants of study expectations, identifying potential barriers, and offering personalized support to facilitate ongoing participation.

Additionally, study activities in the control group are designed to match the intervention group in terms of interaction frequency and engagement level, thereby supporting retention and minimizing the likelihood of differential dropout between conditions. All instances of non-adherence (e.g., failure to complete study activities) and non-retention (e.g., participant dropout) will be systematically documented, along with any reported reasons for disengagement.

### Intervention fidelity

A comprehensive Manual of Operations has been developed to guide all aspects of training, intervention implementation, data transfer, and data management. Standardized protocols have been established for participant screening, enrollment, tracking, and communications. All research staff received training on study procedures, and ongoing team meetings are used to address emerging issues related to data collection, participant engagement, and LMH-4-DCP platform functionality.

**Fig. 2:**
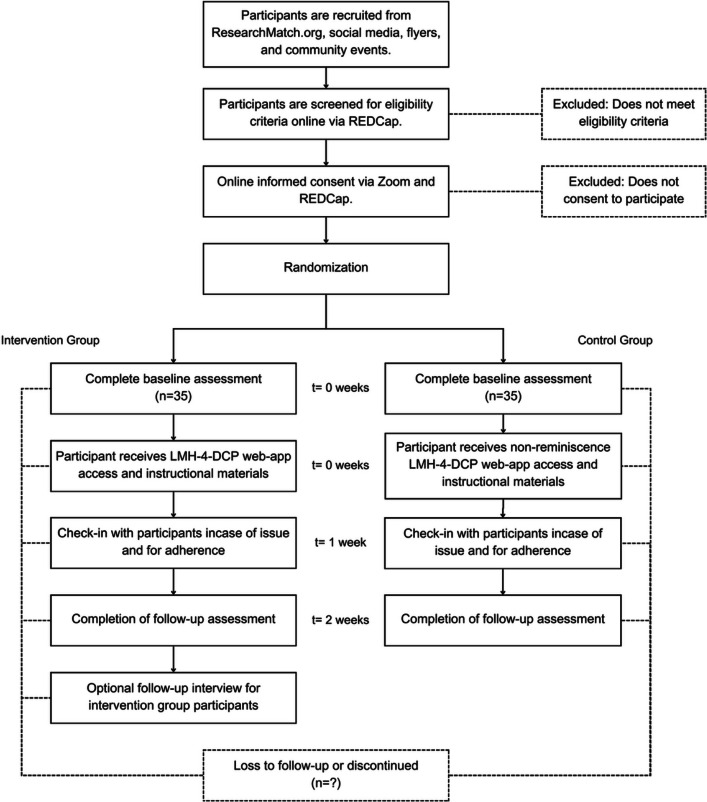
Trial flow diagram

Intervention activities and group assignments are fixed across participants to maintain consistency. All participants follow a standardized 2-week intervention schedule, with data collection at predetermined time points (see Fig. 2 for Trial Flow Diagram). Scripted email templates are used for 1-week check-ins to ensure uniform communication and to address potential concerns or barriers to engagement.

To ensure standardized onboarding, participants receive condition-specific training materials, including PDF guides and short instructional videos tailored to their assigned group. Platform access is controlled through secure, group-specific links, which prevent cross-condition contamination. The LMH-4-DCP interface is identical across participants within a condition, with personalization limited to visual customization of the user’s virtual home environment and activity choices.

### Harms

Adverse events (AEs), defined as any unfavorable or unintended experience associated with study participation, regardless of causality, are anticipated to be rare due to the low-risk, non-invasive nature of the intervention. Potential risks may include emotional discomfort responding to survey questions or intervention-related writing activities, as well as a minimal risk of breach of confidentiality. Should a participant experience emotional distress during the study, a licensed mental health professional from WCM’s Center for Research on End-of-Life Care will be available to conduct further assessment and, if requested, provide referrals to appropriate mental health services.

All AEs and protocol deviations—whether anticipated or unanticipated—will be documented using the study’s designated reporting forms and reviewed at regular intervals as part of continuing review. Events will also be recorded in a centralized study deviation log. In the unlikely event that a medical issue arises that could be construed as serious or requiring urgent follow-up, the study’s multiple principal investigators (MPIs) will be notified within 24 hours, and appropriate institutional reporting procedures will be followed, consistent with IRB and local site policies.

### Monitoring

#### Data monitoring committee

Given the minimal risk associated with study participation, the WCM IRB determined that a formal Data Monitoring Committee (DMC) was not required for this pilot trial. In alignment with institutional oversight policies, the study team is required to submit an annual post-approval monitoring report to the WCM IRB, which includes updates on study progress, protocol adherence, and any unanticipated issues and/or adverse events. This reporting process ensures ongoing compliance with ethical and regulatory standards throughout the study period.

#### Trial monitoring

No interim analyses or stopping guidelines are planned due to the study’s short duration and minimal risk presented to participants. The study team continuously monitors trial progress and data collection to ensure protocol fidelity and protection of human subjects. Any necessary modifications or concerns are reported in accordance with institutional requirements and sponsor expectations.

#### Frequency and plans for auditing trial conduct

There are no plans for auditing trial conduct due to the small-scale nature of the study. However, the IRB and the CTSC reserve the right to audit the study at their discretion.

### Statistical analysis

Descriptive statistics (means, standard deviations, frequencies) will be used to characterize the full sample. Feasibility and acceptability of the LMH-4-DCP intervention will be assessed descriptively by examining recruitment and retention metrics, reasons for refusal, SUS scores, completion rates, and participant-reported usefulness and satisfaction ratings in the intervention group. Pearson’s correlations will be used to assess relationships among continuous variables. Additional analyses will examine associations between outcome measures and potential covariates (e.g., caregiver age, education level, kinship relationship, degree of cognitive impairment in the care recipient). 

To examine preliminary efficacy, within- and between-group differences on secondary outcomes (pre-death grief, relationship quality) will be assessed. Specifically, we will examine (i) mean values at each time point by group, (ii) within-group change from baseline to follow-up, (iii) between-group differences in change, and (iv) standardized effect sizes (e.g., Hedges’ *g*) with 95% confidence intervals to guide planning for a future efficacy trial. Missing data will be handled via listwise deletion. A *p*-value of 0.05 will be used in all analyses.

### Dissemination plans

The final report will be submitted to a peer-reviewed journal for publication to support dissemination to stakeholders across the research–clinical–community continuum, as well as to the general public. Authorship of the final report will be determined by the study’s MPIs and based on individuals’ contributions to the trial. In addition, published results will be translated into lay language and formatted for accessibility to be shared with study participants who express interest and consent for their email addresses to be retained. Individual-level de-identified participant data will be made available upon request after the study-specific aims have been completed and published.

## Discussion

The current study aims to determine the feasibility and acceptability of LMH-4-DCP, a web-based intervention for ADRD care pairs. LMH-4-DCP is designed as a pre-death activity that leverages reminiscence to promote meaningful interpersonal engagement between individuals with ADRD and their caregivers, with the overall goal of strengthening relationship quality and reducing pre-death grief.

The adaptation, design, and development of the intervention were conceptually guided by the Interdependence Model of Communal Coping [20] and the Micro-Sociological Theory of Adjustment to Loss [23]. These theoretical frameworks posit that targeting relationship-based mechanisms can address the complex interpersonal dynamics inherent in ADRD caregiving, thereby reducing pre-death grief and improving relationship quality. A user-centered design approach was also employed to inform the design and refinement of the platform’s interface, ensuring usability, accessibility, and alignment with the needs and preferences of both ADRD family caregivers and PwD. Together, these approaches strengthen the intervention’s design and relevance, increasing its potential for successful implementation in real-world settings.

Data collected on key feasibility indicators—including recruitment, retention, adherence, and participant satisfaction—will provide insight into whether LMH-4-DCP can be effectively delivered in a web-based format and is perceived as useful and usable among the target population. Additionally, exploratory analyses will provide preliminary effect size estimates of LMH-4-DCP on psychosocial outcomes, specifically pre-death grief and relationship quality, thereby establishing critical groundwork for a fully powered efficacy trial.

Findings from this pilot study will inform iterative refinements to the content and delivery of the intervention, as well as guide the design of a larger-scale RCT. If demonstrated to be feasible and acceptable, LMH-4-DCP may offer a scalable and accessible behavioral intervention to support caregiver well-being and preserve relational quality in the context of progressive cognitive decline.

### Trial status

This manuscript was based on protocol version 11.0, dated April 3, 2025; approval received on May 12, 2025. Recruitment for this study began on November 6, 2024. Final enrollment was completed on May 6, 2025. This study protocol has been submitted before the final participant visit, with an estimated study completion date of June 16, 2025.

## Supplementary Information

Supplementary Material 1: S1. SPIRIT Checklist. S2. Original IRB Approval Letter. S3. Current IRB Approval Letter. S4. Informed Consent Form. S5. World Health Organization (WHO) Trial Registration Data Set.

## Data Availability

Not applicable. Data sharing is not applicable to this article as no data were analyzed or generated. Upon completion of the project’s study aims, data will be available upon reasonable request.

## Abbreviations

AE: Adverse events
ADRC: Alzheimer’s Disease Research Center
ADRD: Alzheimer's Disease and Related Dementias
CFI: Comparative Fit Index
CONSORT: Consolidated Standards of Reporting Trials
CTSA: Clinical and Translational Science Award
CTSC: Clinical and Translational Science Center
DMC: Data Monitoring Committee
GRECC: Geriatrics Research Education and Clinical Center
HIPAA: Health Insurance Portability and Accountability Act
ICH-GCP: International Council for Harmonisation of Technical Requirements for Registration of Pharmaceuticals for Human Use on Good Clinical Practice
IRB: Institutional Review Board
ITT: Intent-to-Treat
LDAP: Lightweight Directory Access Protocol
LME: Linear Mixed Effects
LMH: Living Memory Home
LMH-4-DCP: Living Memory Home for Dementia Care Pairs
MPIs: Multiple Principal Investigators
NIH: National Institutes of Health
PG-12: Prolonged Grief 12-item
PwD: Persons with dementia
PGD: Prolonged Grief Disorder
RA: Research assistant(s)
RCT: Randomized controlled trial
REDCap: Research Electronic Data Capture
RT: Reminiscence therapy
RMSEA: Root mean square error of approximation
sIRB: Single IRB
SPIRIT: Standard Protocol Items: Recommendations for Interventional Trials
SUS: System Usability Scale
UNC: University of North Carolina
USA: United States of America
USC: University of Southern California
WCM: Weill Cornell Medicine
