# Supplementary material for: Living Memory Home for Dementia Care Pairs (LMH-4-DCP): study protocol for a pilot randomized trial of a web-based reminiscence intervention for family caregivers and persons with dementia
Source: Pilot Feasibility Stud. 2026 Apr 14;12:79. doi: 10.1186/s40814-026-01781-2 (PMC13191886; doi:10.1186/s40814-026-01781-2)
Supplement: Supplementary file 1 — Supplementary Material 1: S1. SPIRIT Checklist. S2. Original IRB Approval Letter. S3. Current IRB Approval Letter. S4. Informed Consent Form. S5. World Health Organization (WHO) Trial Registration Data Set. [file 40814_2026_1781_MOESM1_ESM.pdf]

Supplementary Materials S.1.: SPIRIT Checklist

SPIRIT 2025 checklist of items to address in a randomized trial protocol

Section / Topic	No	SPIRIT 2025 checklist item description	Reported on page no.
**Administrative information**			
Title and structured summary	1a	Title stating the trial design, population, and interventions, with identification as a protocol	1
	1b	Structured summary of trial design and methods, including items from the World Health Organization Trial Registration Data Set	Supplementary Materials (S.5.)
Protocol version	2	Version date and identifier	23
Roles and responsibilities	3a	Names, affiliations, and roles of protocol contributors	1, 23
	3b	Name and contact information for the trial sponsor	24
	3c	Role of trial sponsor and funders in design, conduct, analysis, and reporting of trial; including any authority over these activities	24
	3d	Composition, roles, and responsibilities of the coordinating site, steering committee, endpoint adjudication committee, data management team, and other individuals or groups overseeing the trial, if applicable	N/A
**Open science**			
Trial registration	4	Name of trial registry, identifying number (with URL), and date of registration. If not yet registered, name of intended registry	23
Protocol and statistical analysis plan	5	Where the trial protocol and statistical analysis plan can be accessed	N/A
Data sharing	6	Where and how the individual de-identified participant data (including data dictionary), statistical code, and any other materials will be accessible	21, 24
Funding and conflicts of interest	7a	Sources of funding and other support (e.g., supply of drugs)	24
	7b	Financial and other conflicts of interest for principal investigators and steering committee members	24
Dissemination policy	8	Plans to communicate trial results to participants, healthcare professionals, the public, and other relevant groups (e.g., reporting in trial registry, plain language summary, publication)	21
**Introduction**			
Background and rationale	9a	Scientific background and rationale, including summary of relevant studies (published and unpublished) examining benefits and harms for each intervention	3
	9b	Explanation for choice of comparator	10
Objectives	10	Specific objectives related to benefits and harms	6
**Methods: Patient and public involvement, trial design**			
Patient and public involvement	11	Details of, or plans for, patient or public involvement in the design, conduct, and reporting of the trial	8

Trial design	12	Description of trial design including type of trial (e.g., parallel group, crossover), allocation ratio, and framework (e.g., superiority, equivalence, non-inferiority, exploratory)	6
**Methods: Participants, interventions, and outcomes**			
Trial setting	13	Settings (e.g., community, hospital) and locations (e.g., countries, sites) where the trial will be conducted	7
Eligibility criteria	14a	Eligibility criteria for participants	7
	14b	If applicable, eligibility criteria for sites and for individuals who will deliver the interventions (e.g., surgeons, physiotherapists)	N/A
Intervention and comparator	15a	Intervention and comparator with sufficient details to allow replication including how, when, and by whom they will be administered. If relevant, where additional materials describing the intervention and comparator (e.g., intervention manual) can be accessed	9
	15b	Criteria for discontinuing or modifying allocated intervention/comparator for a trial participant (e.g., drug dose change in response to harms, participant request, or improving/worsening disease)	17
	15c	Strategies to improve adherence to intervention/comparator protocols, if applicable, and any procedures for monitoring adherence (e.g., drug tablet return, sessions attended)	16
	15d	Concomitant care that is permitted or prohibited during the trial	N/A
Outcomes	16	Primary and secondary outcomes, including the specific measurement variable (e.g., systolic blood pressure), analysis metric (e.g., change from baseline, final value, time to event), method of aggregation (e.g., median, proportion), and time point for each outcome	14
Harms	17	How harms are defined and will be assessed (e.g., systematically, non-systematically)	18
Participant timeline	18	Time schedule of enrollment, interventions (including any run-ins and washouts), assessments, and visits for participants. A schematic diagram is highly recommended (see Figure)	14; Figure 1
Sample size	19	How sample size was determined, including all assumptions supporting the sample size calculation	8
Recruitment	20	Strategies for achieving adequate participant enrollment to reach target sample size	10
**Methods: Assignment of interventions**			
Randomization:			
Sequence generation	21a	Who will generate the random allocation sequence and the method used	13
	21b	Type of randomization (simple or restricted) and details of any factors for stratification. To reduce predictability of a random sequence, other details of any planned restriction (e.g., blocking) should be provided in a separate document that is unavailable to those who enroll participants or assign interventions	13
Allocation concealment mechanism	22	Mechanism used to implement the random allocation sequence (e.g., central computer/telephone; sequentially numbered, opaque, sealed containers), describing any steps to conceal the sequence until interventions are assigned	13
Implementation	23	Whether the personnel who will enroll and those who will assign participants to the interventions will have access to the random allocation sequence	13

Blinding	24a	Who will be blinded after assignment to interventions (e.g., participants, care providers, outcome assessors, data analysts)	13
	24b	If blinded, how blinding will be achieved and description of the similarity of interventions	13
	24c	If blinded, circumstances under which unblinding is permissible, and procedure for revealing a participant's allocated intervention during the trial	13
**Methods: Data collection, management, and analysis**			
Data collection methods	25a	Plans for assessment and collection of trial data, including any related processes to promote data quality (e.g., duplicate measurements, training of assessors) and a description of trial instruments (e.g., questionnaires, laboratory tests) along with their reliability and validity, if known. Reference to where data collection forms can be accessed, if not in the protocol	13-16
	25b	Plans to promote participant retention and complete follow-up, including list of any outcome data to be collected for participants who discontinue or deviate from intervention protocols	16
Data management	26	Plans for data entry, coding, security, and storage, including any related processes to promote data quality (e.g., double data entry; range checks for data values). Reference to where details of data management procedures can be accessed, if not in the protocol	12
Statistical methods	27a	Statistical methods used to compare groups for primary and secondary outcomes, including harms	19
	27b	Definition of who will be included in each analysis (e.g., all randomized participants), and in which group	19
	27c	How missing data will be handled in the analysis	20
	27d	Methods for any additional analyses (e.g., subgroup and sensitivity analyses)	20
**Methods: Monitoring**			
Data monitoring committee	28a	Composition of data monitoring committee (DMC); summary of its role and reporting structure; statement of whether it is independent from the sponsor and funder; conflicts of interest and reference to where further details about its charter can be found, if not in the protocol. Alternatively, an explanation of why a DMC is not needed	18
	28b	Explanation of any interim analyses and stopping guidelines, including who will have access to these interim results and make the final decision to terminate the trial	19
Trial monitoring	29	Frequency and procedures for monitoring trial conduct. If there is no monitoring, give explanation	19
**Ethics**			
Research ethics approval	30	Plans for seeking research ethics committee/institutional review board approval	11
Protocol amendments	31	Plans for communicating important protocol modifications to relevant parties	11
Consent or assent	32a	Who will obtain informed consent or assent from potential trial participants or authorized proxies, and how	11
	32b	Additional consent provisions for collection and use of participant data and biological specimens in ancillary studies, if applicable	N/A
Confidentiality	33	How personal information about potential and enrolled participants will be collected, shared, and maintained in order to protect confidentiality before, during, and after the trial	12

Ancillary and post-trial care	34	Provisions, if any, for ancillary and post-trial care, and for compensation to those who suffer harm from trial participation	18

Citation: Chan A-W, Boutron I, Hopewell S, Moher D, Schulz KF, et al. SPIRIT 2025 statement: updated guideline for protocols of randomised trials. BMJ 2025;389:e081477. https://dx.doi.org/10.1136/bmj-2024-081477

© 2025 Chan A-W et al. This is an Open Access article distributed under the terms of the Creative Commons Attribution License (https://creativecommons.org/licenses/by/4.0/), which permits unrestricted use, distribution, and reproduction in any medium, provided the original work is properly cited.

Supplementary Materials S.2.: Original IRB Approval Letter

Weill Cornell Medicine
Research Integrity

Institutional Review Board

Notification of Approval

17-Oct-2023

To: Holly Prigerson PhD

Study Title: The Living Memory Home: Reducing Grief and Improving Relationships between Home-based Patients with ADRD and Their Family Caregivers

**WRG Submission Number**	23-07026251-02
**Approval Date**	16-Oct-2023
**Expiration Date**	n/a
**Post Approval Monitoring Due**	15-Oct-2024
**Risk Level**	Minimal Risk
**FWA Number**	FWA00000093

This protocol was reviewed and approved by the WCM IRB in accordance with ethical standards and the requirements of the Code of Federal Regulations on the Protection of Human Subjects (45 CFR 46, and if applicable 21 CFR 50, 56, 312, and 812).

The following determinations have been made:

This protocol was approved under expedited categories 6 and 7.

Informed Consent: must be obtained and documentation is required.

HIPAA Authorization: is required for this project.

These determinations apply only to the activities described in the submission and may not apply should any changes be made to this research.

Modifications or changes to this protocol must be submitted to the WCM IRB for review and approval prior to implementation. Once all research activities have been completed, a request for closure must be submitted.

As part of the WCM IRB's Post Approval Monitoring program, a Post-Approval Monitoring Annual Report (PAM-AR) will be required annually. Please submit your post-approval monitoring report at least 60 days before the due date listed above.

The principal investigator must report any adverse events or unanticipated problems resulting from this study to the WCM IRB. The principal investigator remains responsible for following all pertinent ethical and legal guidelines as well as WCM policies.

In order to access the approved docs tab, click on the Submission Page. The Approved Documents tab will appear on the top bar next to the Linkages and Submissions tab.

If you have any questions, please contact Human Research Compliance at 646-962-8200 or email irb@med.cornell.edu.

Sincerely,

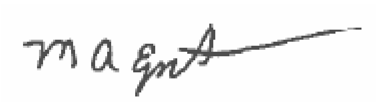

Melissa A. Epstein, PhD, MBE, CIP

Executive Director

Human Research Protection and Compliance

Weill Cornell Medicine

This is official correspondence from the WCM IRB Office

research.weill.cornell.edu

Supplementary Materials S.3.: Current IRB Approval Letter

Weill Cornell Medicine
Research Integrity

Institutional Review Board

Notification of Approval - Amendment

12-May-2025

To: Holly Prigerson PhD

Amendment to Study Title: The Living Memory Home: Reducing Grief and Improving Relationships between Home-based Patients with ADRD and Their Family Caregivers

**WRG Submission Number**	23-07026251-15
**Approval Date of Amendment**	12-May-2025
**Protocol Expiration Date**	n/a
**Post Approval Monitoring Due**	18-Aug-2025
**Risk Level**	Minimal Risk
**FWA Number**	FWA00000093

This amendment was reviewed and approved by the WCM IRB in accordance with ethical standards and the requirements of the Code of Federal Regulations on the Protection of Human Subjects (45 CFR 46, and if applicable 21 CFR 50, 56, 312, and 812).

Additional modifications or changes to this protocol must be submitted to the WCM IRB for review and approval prior to implementation. Once all research activities have been completed, a request for closure must be submitted.

As part of the WCM IRB's Post Approval Monitoring program, a Post-Approval Monitoring Annual Report (PAM-AR) will be required annually. Please submit your post-approval monitoring report at least 60 days before the due date listed above.

The principal investigator must report any adverse events or unanticipated problems resulting from this study to the WCM IRB. The principal investigator remains responsible for following all pertinent ethical and legal guidelines as well as WCM policies.

In order to access the approved documents tab, click on the Submission Page: Submission Page. The Approved Documents tab will appear on the top bar next to the Linkages and Submissions tab.

If you have any questions, please contact Human Research Compliance at 646-962-8200 or email irb@med.cornell.edu.

Sincerely,

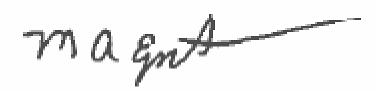

Melissa A. Epstein, PhD, MBE, CIP
Executive Director
Human Research Protection and Compliance
Weill Cornell Medicine

This is official correspondence from the WCM IRB Office

research.weill.cornell.edu

Weill Cornell Medicine
Research Integrity

Supplementary Materials S.4.: Informed Consent Form

Research Consent

The Living Memory Home for Dementia Care Pairs (LMH-4-DCP): Reducing Grief and Improving Relationships between Home-Based Patients with ADRD and Their Family Caregivers (PHASE 2)

April 3rd, 2025

Please read this form or have this form read to you. Take your time to make your decision. Make sure we have explained the study to you. Ask us any questions. You may also want to talk about the study with your doctor, family, loved ones, or friends. The choice to join the study, or not, is yours. If you decide to join, please sign and date this form.

Why is this study being done?
You are being asked to join Phase 2 of a research study funded by the National Institute on Aging (NIA) led at Weill Cornell Medicine by Principal Investigators Holly G. Prigerson, PhD and Francesca Falzarano, PhD. The purpose of this study is to conduct a randomized-control trial (RCT) to examine the feasibility and acceptability of the Living Memory Home for Dementia Care Pairs (LMH-4-DCP) web application, a pre-loss reminiscence-based platform for caregivers and persons with dementia (PwD) in the mild-to-moderate stages of the disease course. We also aim to explore the potential for LMH-4-DCP to improve psychosocial well-being. You are being asked to join because you are

A. A **family caregiver** who 1) is a family member or friend who provides the majority of care for a person in the early to moderate stages of Alzheimer's disease or related dementias (ADRD); 2) is over the age of 18-years-old; 3) is English-speaking; 4) is able to use the internet and has internet access at home; 5) resides in the United States.

Important information for you to think about:

- **What am I being asked to do?** After completing all screening and eligibility measures as well as providing your informed consent, you will be emailed a secure REDCap link to complete a battery of online surveys as part of your baseline assessment. Then, the caregiver will be given access to and instruction on how to use LMH-4-DCP. During the two-week intervention, caregivers will lead care-recipients in helping to complete assigned study activities. You will receive instructional training on how to interact with the application prior to your first login. You will be asked to log-in to the application three times per week for the two-week study duration. You will then complete a post-intervention follow-up assessment and be invited for an optional feedback interview.

- **How long will the study last?** If you join the research study, participation will last approximately one-month. Following completion of your baseline assessment, you will be asked to interact with the web application for two weeks and encouraged to log in alongside your caregiver or care recipient at least 3 times per week.
- **Any possible risks or discomforts?** Potential risks are minimal. Participants may experience distress or discomfort related to the content of study activities, as you reflect on the ADRD experience and recall past memories. However, you may stop participating at any time without penalty
- **Will this study help me?** While we cannot guarantee that you will benefit directly from this study, your contribution is significant in helping the study team examine the potential for a web-based reminiscence platform to enhance well-being in dementia care pairs.
- **Do I have to join?** If you decide to join this study, it should be because you want to volunteer. You will not lose any services, benefits or rights, or access to care you would normally have if you chose not to volunteer.

If you are now or have been (within the last 6 months) in any other research study, please tell the research team.

What will I be asked to do?
First, the family caregiver will complete a self-report online survey with questions regarding their background and demographic characteristics (e.g., age, gender, race, ethnicity, marital status), which will take 5-10 minutes to complete. They will also be asked to answer several questions about the person(s) with dementia they provide care for (e.g., dementia type/severity, hours of care provided per week), including questions regarding whether the caregiver thinks the PwD would benefit from the study. Once the caregiver completes this self-reported online survey, a member of the study team will contact the family caregiver to tell them of their eligibility status to participate.

Once consent from the caregiver has been obtained, a member of the study team will email the caregiver a link to complete the baseline assessment, which includes an online survey battery of measures assessing your levels of social support, quality of life, and experience with technology. After completing the baseline assessment, and randomization, you will receive training materials that include how to access and use the web application.

You will be randomly assigned to one of two study groups. This randomization process is like flipping a coin, assigning you to a group by chance. Neither you nor the researchers will have any influence over the group selection. You will either be assigned to the intervention group, which includes a version of the web application with reminiscence-based therapy features and activities (LMH-4-DCP), or the control group, which includes a version of the web application without the reminiscence-based therapy features. You will receive specific training materials tailored to your group assignment that will provide step-by-step instructions on how to access and use the website.

Both versions of the web application will invite the caregiver to work with their PwD to create a virtual home where the care pair will come together and engage in activities and features specific to the caregiver’s assigned study group. Because the application is designed for wide-screen devices, the web application must be accessed from a laptop, tablet, or desktop computer. *We ask that you*

log-in to the web application at least three times per week to complete study assignments across the two-week intervention duration, for a total of six log-ins.

One week after you gain access to the web application, a member of the study team will email you to confirm your use of the application and address any questions or concerns you may have. A call may be scheduled as well if you would like.

After using LMH-4-DCP for two weeks, you will then complete another series of surveys as your follow-up assessment. The follow-up survey battery will include the same measures administered to you at baseline in addition to providing feedback on your experience engaging with the web application. Upon completion of study activities, participants will be provided with the opportunity to engage in an optional follow-up semi-structured interview to elaborate on their experiences with and feedback on the web application.

Do I have other choices?
Instead of being in this study, you can choose not to participate in this study.

Who will be in the study?
Approximately 70 family caregivers will be enrolled in this study.

New information that may change your decision to join:
During the study, we will tell you if there is new information or changes to the study that could affect you, your health, or your desire to continue your participation in the study. You will be notified in a timely manner if important new findings become known that may affect your willingness to continue in the study.

What are the risks?
Participating in a research study can pose risks such as stress, emotional discomfort, inconvenience, and potential breaches of privacy and confidentiality. However, the risks involved with participation in this study are considered minimal. They include a small risk of loss of confidentiality of data and possible discomfort when answering sensitive questions and reflecting on your experiences related to dementia or caregiving for someone with dementia.

Can being in this study help me?
While we cannot guarantee that you will benefit directly from this study, however, your participation will contribute to the development and refinement of a feasible, acceptable web-based reminiscence platform to improve psychosocial well-being among dementia caregivers.

Can being in this study benefit the researchers or WCM/NYP?
The researchers of this study do not receive any direct benefit from this study.

How will my information be used and protected?
Efforts will be made to protect your medical records and other personal information to the extent allowed by law. We will take steps to help make sure that all the information we get about you is kept confidential. Your name will not be associated with any of your study responses and will be assigned a unique identifier number (described in further detail below), however, we cannot guarantee absolute confidentiality. Records of study participants are stored and kept according to legal requirements. You will not be identified personally in any reports or publications resulting from this study. Organizations that may request to inspect and/or copy your research and medical records for quality assurance and data analysis include groups such as:

- Weill Cornell Medicine Principal Investigator and study team
- The WCM Institutional Review Board (IRB)

- The Office of Human Research Protection (OHRP)
- Department of Health and Human Services
- National Institutes of Health (NIH)/National Institute on Aging (NIA)

By signing this consent form, you authorize access to this confidential information.

We will take the following precautions to protect subject data being stored on a computer from unauthorized disclosure, tampering, or damage by requiring a unique ID and password to log into the database. Individuals who enroll in the study will be assigned a unique identifier number to which their data will be linked. A document linking study identifier numbers to identifying information will be stored in a single master document on a secure WCM server that can only be accessed via password-protected computers that are stored in locked offices only accessible by credentialed study personnel. All coded data will be stored on a secure WCM server that can only be accessed by credentialed study personnel on password protected computers in locked offices. All audio files will be destroyed once a text transcription of interview sessions is complete. In addition, only personnel who are associated with the study will have access to the study specific records in the database.

A Certificate of Confidentiality has been granted by the Department of Health and Human Services (DHHS). This Certificate will protect the investigators (research/study staff) from being forced to release any research data in which the subject is identified even under a court order or subpoena. This protection is not absolute. For instance, it does not override any state requirement to report child abuse to the appropriate authorities.

A description of this clinical trial will be available on http://www.ClinicalTrials.gov. This website will not include information that can identify you. At most, the website will include a summary of the study results. You can search this website at any time.

Will my information be used in the future?
We will destroy information about you when the study is finished. Information about you will only be kept for as long as required by regulations and WCM policy and will not be used or shared for future studies unless you provide your consent to retain this information.

Will I be paid?
Each participant will be compensated with $100 for their completed study participation. Compensation will be provided to the caregiver in two $50 payments, which will be provided to participants upon completion of the baseline and follow-up assessments, respectively. This compensation will be sent via email in the form of an Amazon digital gift card. If you choose to participate in the optional interview, you will receive an additional $20 Amazon digital gift card.

Do I have to join? Can I quit the study?
It is your decision whether to join this study or not. You have the right to choose not to join, to skip any questions you may be uncomfortable answering, or to stop your participation at any time. Your decision to join in this research or stop participating will not affect your regular care nor your relationship with Weill Cornell Medicine, your doctors, or other employees. The research staff will discuss with you any requested processes (e.g., final study visit, etc.) needed for your safe withdrawal from the study. If you decide to discontinue your participation, please let a member of the study team know.

Your participation in this study may be stopped without your consent at any time and for any reason by the sponsor, the FDA and/or other regulatory agencies. Reasons you may be

withdrawn from the study include it is determined to be in your best interest, you need treatment not allowed in this study, you do not follow the study instructions, or for other administrative reasons.

After you leave the study, no new information will be collected from you. Information that has already been collected can be withdrawn from the database if you choose. You will be told about new information that may affect your health, wellbeing, or participation in this study.

What are the costs?
There are no direct costs from being in this research study.

Will I get the study results?
Published results of this study will be provided to you following its completion unless you opt out.

Will I be able to see or copy my information?
During this study, you will be able to see or receive a copy of your protected health information as described in this form per Weill Cornell Medicine policies. During your participation in this study, you will have access to your research record and any study information that is part of that record. To request your research record, you can email sek4011@med.cornell.edu.

What if I have questions?
If you have any questions, concerns, or complaints about the research, please contact:

Holly Prigerson, PhD & Francesca Falzarano, PhD
Cornell Center for Research on End-of-Life Care
420 East 70th Street
4th Floor, LH 401-407
New York, NY 10021
hgp2001@med.cornell.edu

If you have questions regarding your rights as a research participant, about what you should do in case of any injury or illness because of your study participation, or if you want to get information or give feedback, please contact the WCM Institutional Review Board (IRB) at:

WCM IRB, (646) 962-8200, irb@med.cornell.edu

Website: https://research.weill.cornell.edu/irb

You may also submit questions or complaints without giving your name by calling (866) 293-3077 or visiting http://www.hotline.cornell.edu/.

HIPAA Authorization for Use and Disclosure of Your Protected Health Information
As part of this study, we will be collecting health information about you and sharing it with others. This information is “protected” because it identifies you.

Protected Health Information (PHI)
By signing this Consent Document, you are allowing the following people to use or release your protected health information for this study. Weill Cornell Medicine researchers need your permission to use your protected health information according to a regulation called the HIPAA (Health Insurance Portability & Accountability Act) Privacy Rule.

Voluntary Choice: The choice to give Weill Cornell Medicine researchers permission to use or share your protected health information for their research is voluntary. It is completely up to you. No one can force you to give permission. However, you must give permission for Weill Cornell Medicine researchers to use or share your protected health information if you want to participate in the study. If you decline to sign this form, you cannot participate in this study, because the researchers will not be able to obtain and/or use the information they need to conduct their research. Refusing to give permission will not affect your ability to get usual treatment, or health care from Weill Cornell Medicine.

Protected Health Information to Be Used or Shared: Government rules require that researchers get your permission (authorization) to use or share your protected health information. Your medical information may be disclosed to authorized public health or government officials for public health activities when required or authorized by law. If you grant permission, the researchers may use or disclose any protected health information related to this research study from your survey data to the entities mentioned above on page 4. *We will collect basic identifying information for all participants who indicate an interest in participating in the study, including name, address, phone number, and email. These details will be used for contacting participants, screening for eligibility, and arranging a time to obtain informed consent. Individuals enrolled in this study will receive a unique identifier number. Participant's identifying information will be stored separately from the study data in a distinct database to ensure confidentiality.*

Other Use and Sharing of Protected Health Information: If you give permission, the researchers could also use your protected health information to develop new procedures or commercial products. They could share your protected health information with the study sponsor, the WCM Institutional Review Board, inspectors who check the research, government agencies and research study staff.

The information that may be shared with the sponsor and/or government agencies could include your research record related to this study. They may not be considered covered entities under the Privacy Rule and your information would not be subject to protections under the Privacy Rule. We will use and share your information only as described in this form; however, people outside WCM who receive your information may not be covered by this promise or by the federal Privacy Rule. We try to make sure that everyone who needs to see your information keeps it confidential – but we cannot guarantee that your information will not be re-disclosed.

In addition to the people listed in this form, there is a chance that your health information may be shared outside of the research study and no longer be protected by federal privacy laws. Examples of this include releases to law enforcement, legal proceedings, health oversight activities and public health measures.

Right to Withdraw Your Authorization
Your permission for the use and disclosure of your health information for this project shall not expire unless you cancel it. However, you may stop your permission at any time by notifying the WCM Privacy Office in writing. To do this, please send a letter to:

Privacy Office
1300 York Avenue, Box 303
New York, NY 10065

Email: privacy@med.cornell.edu

If you have questions about this and would like to discuss them, please call (646) 962-6930. Please note that the research team does not have to destroy or retrieve any of your health information that has already been used or shared before your withdrawal is received.
If you have questions about the privacy practices of the institution, you can request a Notice of Privacy Practices from your provider.

Refusal to Sign
If you choose not to sign this consent form and permission for the use and disclosure of your PHI, you cannot be in the research study. Your decision to sign this consent form or stop participating will not affect your regular care, benefits, nor your relationship with Weill Cornell Medicine, NYP, your doctors, or other employees.

Signature

I agree to participate in this research and allow my information to be used in this research. My questions have been answered. I will get a signed copy of this form.

____________________________	_______________________________	________
Name of Adult Participant	Signature of Adult Participant	Date

Audio Recording Signature

Please check one of the following:

☐ I agree to be audio recorded.
☐ I do not want to be audio recorded.

Researcher Signature (to be completed at time of informed consent)

I confirm that the research study was thoroughly explained to the participant. I reviewed the consent form and answered all questions. The participant appeared to have understood the information.

____________________________	______________________________	________
Name of Research Team Member	Signature of Research Team Member	Date

Supplementary Materials S.5.: World Health Organization (WHO) Trial Registration Data Set

1. Primary registry and trial identifying number: Clinicaltrials.gov: NCT06225986

2. Date of registration in primary registry: January 9, 2024

3. Secondary identifying numbers (if applicable): NIA R21AG077144 [U.S. NIH Grant/Contract Award Number]

4. Source(s) of Monetary or Material Support: National Institute on Aging (NIA)

5. Primary Sponsor: Weill Medical College of Cornell University

6. Secondary Sponsor(s) (if any): Not applicable

7. Contact for Public Queries: Sydney C. Saviano, B.A., Email: sys4002@med.cornell.edu
   Holly G. Prigerson, Ph.D., Email: hgp2001@med.cornell.edu
   Francesca B. Falzarano, Ph.D., Email: falzaran@usc.edu

8. Contact for Scientific Queries: Holly G. Prigerson, Ph.D., Email: hgp2001@med.cornell.edu
   Francesca B. Falzarano, Ph.D., Email: falzaran@usc.edu

9. Public Title: Living Memory Home: A digital program to help people with dementia and their family caregivers reduce grief and improve relationships.

10. Scientific Title: Living Memory Home for Dementia Care Pairs (LMH-4-DCP): Pilot randomized feasibility study protocol for a digital intervention to reduce pre-loss grief and improve relationship quality among persons with dementia and their family caregivers

11. Countries of Recruitment: United States

12. Health Condition(s) or Problem(s) Studied: Dyadic family caregiver-person with dementia online intervention

13. Intervention(s):
    - Experimental: Participants access the full LMH-4-DCP website, featuring reminiscence therapy activities (e.g., interactive memory-sharing modules); 3 times per week for 2 weeks (n=35).
    - Attention Control Condition: Participants access a limited version of the LMH-4-DCP platform that excludes reminiscence therapy features and engage in non–reminiscence-based activities (e.g., journal prompts related to present events), 3 times/week for 2 weeks (n=35).

14. Key Inclusion and Exclusion Criteria:
    - Ages eligible for study: ≥18 years
    - Sexes eligible for study: both
    - Accepts healthy volunteers: yes
    - Inclusion criteria: Adults (≥ 18 years) serving as primary source of care persons with mild-to-moderate Alzheimer's disease or Related Dementias (ADRD); fluent in English; Resides in the United States; Has internet access

- Exclusion criteria: Minors (< 18 years) and/or does not serving as primary source of care persons with mild-to-moderate Alzheimer's disease or Related Dementias (ADRD) and/or is not fluent in English, and/or does not reside in the United States, and/or does not have internet access.

15. Study Type (e.g., interventional, observational, etc.): Interventional
    - Allocation: Randomized
    - Intervention Model: Parallel assignment
    - Masking: Yes (Participant)
    - Primary Purpose: Supportive Care
    - Type: Behavioral (e.g., Psychotherapy, Lifestyle Counseling
    - Study Phase: Phase 1

16. Date of First Enrollment (anticipated or actual): November 16, 2024 (Actual)

17. Target Sample Size: N=70 family caregivers

18. Recruitment Status: Recruitment completed May 6, 2025

19. Primary Outcome(s):
    - **Feasibility as measured by recruitment, retention, and system usage metrics**: Feasibility will be assessed through several indicators: (1) Recruitment feasibility will be measured by the number and proportion of family caregivers who express interest, consent to participate, decline participation, or are deemed ineligible at screening; (2) Retention will be assessed by calculating the proportion of participants who complete both baseline and 2-week assessments; and (3) Website feasibility will be evaluated using backend analytics (e.g., number of logins, time spent in features) and a modified 10-item System Usability Scale (SUS). SUS items are rated on a 5-point Likert scale and converted to a 0-100 score, with higher scores indicating greater usability.
        - **Time Frame**: Baseline, 2 Weeks
    - **Acceptability as measured by user experience survey and qualitative feedback**: Acceptability will be assessed at follow-up using an 8-item survey measuring satisfaction, perceived usefulness, and ease of use of the LMH-4-DCP platform. Each item is rated on a 5-point Likert scale (1 = Strongly Disagree to 5 = Strongly Agree), with higher scores indicating greater acceptability. In addition, participants in the intervention group will respond to five open-ended questions to provide qualitative feedback on user experience, including strengths and suggested improvements.
        - **Time Frame**: 2 Weeks

20. Key Secondary Outcome(s):
    - **Change in Grief Severity as measured by the Prolonged Grief-12-Revised (PG-12-R)**: Caregivers' pre-loss grief assessed using the validated 12-item PG-12-R instrument; Scores ranging from 12-60, with higher scores indicative of greater feelings of pre-loss grief.
        - **Time Frame**: Baseline, 2 Weeks
    - **Change in Relationship Quality as measured by three validated instruments:** Relationship quality between caregivers and care-recipients will be assessed using: (1) the 6-item Relational Deprivation Scale, which evaluates perceived relational loss due to the care-recipient’s illness; scores range from 1-4, with higher scores indicating poorer relationship quality; (2) the 15-item Mutuality Scale, which measures the positive aspects of caregiving relationships, with responses from 0-4 and higher scores reflecting greater mutuality; and (3) the 8-item

Relationship Rewards Scale, which assesses perceived relationship quality before and after the ADRD diagnosis, with items rated from 1-4 and higher scores indicating greater relational rewards.

- **Time Frame:** Baseline, 2 Weeks

21. Ethics Review:
    - Status: Approved
    - Date of primary approval: October 16, 2023
    - Date of most recent approval: May 12, 2025
    - Name and contact details of Ethics committee(s):
      Name: Weill Cornell Medicine Institutional Review Board
      Address: 1300 York Avenue, Box 89 New York, NY 10065
      Telephone: 646-962-8200

22. Completion date: Not yet completed

23. Summary Results: Not applicable as the study has not been completed yet.

24. IPD Sharing Statement: Data sharing is not applicable to this article as no data were analyzed or generated. Upon completion of the project's study aims, data will be available upon reasonable request.
